# Design and Performance of a Novel Scalable Core–Sheath Inverted Nozzle Soft Material Pressure Spinner

**DOI:** 10.1021/acspolymersau.5c00174

**Published:** 2026-01-14

**Authors:** Hettiyahandi Binodh De Silva, Angelo Delbusso, Yanqi Dai, Merve Gultekinoglu, Shervanthi Homer-Vanniasinkam, Mohan Edirisinghe

**Affiliations:** 1 Department of Mechanical Engineering, 4919 University College London, London WC1E 7JE, U.K.; 2 Department of Nanotechnology & Nanomedicine, Institute for Graduate Studies in Science & Engineering, Hacettepe University, Ankara 06800, Turkey

**Keywords:** alginate, biopolymers, core−sheathed fibers, core−sheathed inverted nozzle pressurized gyration, natural polymers, spinning, sustainability

## Abstract

Core–sheathed inverted nozzle pressurized gyration (CsINPG) is a novel fiber manufacturing process based on gas blowing-assisted rotary coaxial spinning technology, capable of large-scale manufacture of core–sheathed, micropolymeric structures. The CsINPG spinning vessel is constructed from polycarbonate and has a unique nozzle arrangement, which increases uniformity and facilitates the formation of core–sheathed fibers. The CsINPG apparatus functions as a jet generator, ejecting the spinning feedstock under the combined forces of centrifugal force and pressure differentials. The centrifugal force, which is generated by the spinning of the vessel, is powered by a connected electrical motor. This enables the loaded polymeric feedstock to overcome its surface tension, facilitating fluid ejection through the external nozzles on the vessel wall to form spinning jets. These polymeric jets undergo further stretching through the assimilation of the pressure differential, which is powered by introducing nitrogen flows. This further increases the initial velocity and acceleration. In core–sheathed pressurized gyration, the feedstock is present in two different chambers of the core and the sheath. Furthermore, during “inverted” nozzle-pressurized gyration, the entire manufacturing process is carried out on a horizontal axis, facilitating the controlled streaming of these spinning jets into a water bath. This facilitates the usage of “green polymers” such as alginate and cellulose, which require water baths to be converted from soluble streams to insoluble fibrous structures. These fabricated core–sheathed fibers, manufactured under the optimum parameters in this study, produced fibers with average diameters measuring <10 μm. This paper will delve into the development of the novel CsINPG manufacturing process, focusing on the design of the spinning vessel, the parameters used, the optimization of parameters and their consequences, and the potential future applications of the manufactured core–sheathed fibers.

## Graphical abstract

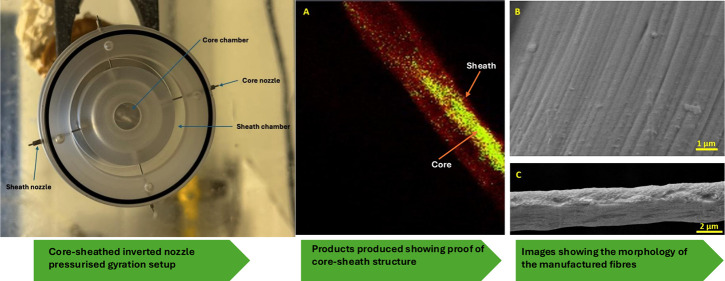

## 1 Introduction

Polymeric materials have become an integral part of human life, encompassing a wide range of goods, ranging from everyday appliances, such as plastic utensils and clothing, to PVC pipes and insulation foams used in the construction industry, as well as advanced polymeric composites, including carbon fiber-reinforced plastics, utilized in the aerospace, aviation, and automotive industries.^1^ However, most of these polymeric materials used by humans are petroleum or fossil fuel-based, which represents a nonrenewable resource. This has given rise to a number of issues, including resource depletion, greenhouse gas emissions leading to climate change, and environmental pollution due to nonbiodegradability. This, in turn, results in the addition of microplastics leading to ocean pollution, which causes further issues such as toxicity and health risks due to bioaccumulation, waste management, and increases in economic costs. These costs must be mitigated.^2^ The extent of damage is visible through the correlation of a 200-fold increase in plastic manufacturing from 2 million tonnes in 1950 ^3^ to 400.3 million tonnes in 2022,^4^ which results in an estimated emission of 10–40 million tonnes of microplastics into the environment.^5^ In the present, microplastics make up 60–80% of all marine debris with 90% of floating oceanic wastes being plastics or other fossil fuel-based products.^6^ This plastic pollution has severe economic consequences, with damages from plastic pollution causing an estimated increase in costs of US$18.3–158.4 trillion from 2016 to 2040.^7^

However, these issues could be overcome by switching to the use of green, natural polymers. These macromolecules are derived from renewable biological sources and offer sustainability, biodegradability, nontoxicity, and biocompatibility, making them environmentally benign throughout their lifecycle.^8^ Examples include polysaccharides such as alginate, cellulose, starch, and chitin, as well as proteins such as casein and gelatin.^9^ Despite the numerous benefits of utilizing green polymers, converting them into commercially valuable products poses a significant challenge. This is due to various factors, including insolubility in popular commercial solvents and complex solvent removal processes resulting from the extreme hydrophilic characteristics of each polymer, as well as the varying molecular weights and branching structures of the polymers. These factors affect overall solubility and complicate the extraction, utilization and conversion of these green polymers into commercially valuable products.^10^ To overcome these challenges, a sustainable and cost-effective manufacturing process was required, which paved the way for the discovery and development of novel fiber manufacturing methods.

Various fiber manufacturing processes have been developed over time, each with its unique advantages and disadvantages, leading to further improvements and discoveries. The development of fiber fabrication methods has followed a clear evolutionary trajectory, with each successive technique addressing the shortcomings of its predecessors while introducing new capabilities. The earliest method of fiber fabrication was phase separation. This technique, although simple and effective for generating porous fibrous structures, suffered from significant limitations, such as poor control over fiber diameter, time-intensive processing, and the risk of solvent retention within the matrix.^11^ In response to the need for improved control and structural uniformity, template synthesis was discovered as a solution. This method enabled precise regulation of fiber dimensions through the use of structured molds, which produced highly uniform fibers. However, the requirement for constant template removal and the lack of scalability hindered its widespread application.^12^ Building on these limitations, self-assembly was developed, introducing the concept of molecular-level organization and facilitating the formation of nanostructures through noncovalent interactions. This approach offered unprecedented control at a nanoscale, proving useful in biomedical applications such as targeted drug delivery. However, slow kinetics and scalability challenges hindered its widespread use.^13^ A significant evolutionary leap occurred with the introduction of electrospinning, which harnessed high-voltage electrostatic forces to produce ultrafine fibers from a wide range of materials. Electrospinning addressed the need for fine diameter control and material versatility but was limited by its low production rate, dependence on high-voltage systems, and the use of volatile solvents.^14^ Recognizing the demand for scalable, high-output fiber production mechanisms led to the development of pressurized gyration (PG). This technique utilizes centrifugal force and gas pressure to increase throughput while maintaining precise control over fiber formation. PG eliminated the need for electrical fields and demonstrated compatibility with various polymers, though it still suffered from a broad fiber diameter distribution and some solvent limitations.^15^ Further refinement of this process occurred with nozzle-pressurized gyration, which introduced a convergent nozzle to focus and direct fiber jets, resulting in more consistent fiber alignment and reduced variability. This was then followed by the inverted nozzle pressurized gyration, fabrication process, which leveraged gravitational assistance to enhance uniformity and directional control during fiber collection. These modifications improved control and consistency but were capable of producing only solid, single-layered fibers.^16^ The most advanced stage in this evolutionary sequence is represented by CsINPG. This technique integrates a coaxial nozzle system into the inverted pressurized gyration setup, enabling the fabrication of core–sheath structured fibers. Such fibers offer multifunctionality, enabling the encapsulation of sensitive agents within a protective shell, making them highly suitable for biomedical, pharmaceutical, and functional material applications. This novel fabrication process exemplifies the convergence of precision, functionality, and scalability, marking a pivotal point in the evolution of fiber manufacturing technologies. This progressive refinement across techniques reflects a broader shift from basic structural generation toward highly controlled, application-driven fiber fabrication, where precision, throughput, and functionality are increasingly optimized. In the present study, the basic mechanism of the CsINPG technique, its optimization stages, its compatibility with natural and environmentally friendly materials, and its potential applications have been examined.

## 2 Experimental Details

### 2.1 Materials

Polycarbonate was obtained from the Ensinger Group, Germany. The tubing for the nozzles was purchased from McMaster-Carr in the United States. The sodium alginate powder (Na-Alg, CAS: 9005-38-3) was purchased from Scientific Laboratory Supplies (UK). General-purpose calcium chloride granules (CaCl_2_, CAS: 10043-52-4) were purchased from Fisher Scientific (UK). The dyes rhodamine B (CAS: 81-88-9) and acriflavine hydrochloride (CAS: 8063-24-9) were purchased from Sigma-Aldrich. Deionized water was used as the solvent.

### 2.2 Vessel Design

The primary inspiration for the development and design of the novel CsINPG pot was obtained through careful analysis and study of the inverted nozzle pressurized gyration pot developed by Dai et al.^17^ and core–sheathed pressurized gyration pots developed by Majd et al.^18^ and Mahalingam et al.^19^ with the physics behind the process comprehended using Alenezi et al.^20^ Studying these pots provided the fundamental understanding and foundation for developing the novel CsINPG setup, facilitating an improved combination of the two manufacturing processes, which led to the development of the novel spinning vessel.

First, as demonstrated in Figure 1, the spinning vessel resembles previous gyration vessels, with a total volume of 60 mL, which is the standardized size for lab-scale gyration vessels. The core chamber has a volume of 10.15 cm^3^, and the sheath chamber has a volume of 35.22 cm^3^, with disparities arising from differences in wall thickness. These chambers’ volumetric capacity facilitates loading 7–8 mL of stock into each chamber during a spinning cycle to form fibrous patches with an estimated production rate of 0.0078 g/s. The chambers are individually filled with stock, and then the lid is screwed on to create an airtight space. This is followed by the pot being mounted onto the clamp. The gas tubing is connected to the nozzle inlet in the center of the lid, and the DC motor, attached to the back of the pot, is connected to the main power supply via crocodile clips. The water bath is placed on a stand with an adjustable knob, allowing for easy height adjustment and collection distance adjustment. Furthermore, the pot was constructed from transparent polycarbonate with a density of 1.19 g/cm^3^.

**Figure 1:**
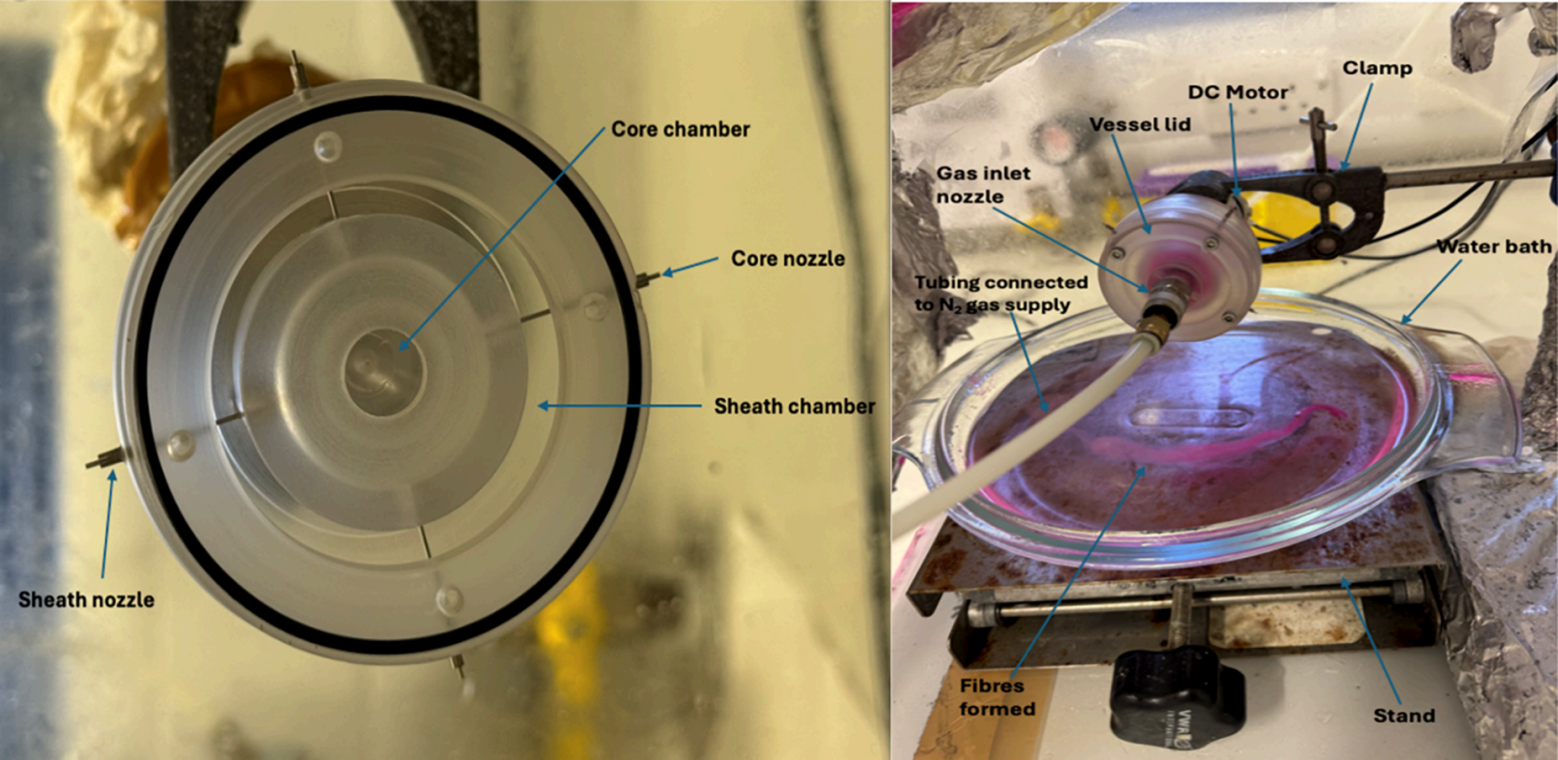
Detailed diagram showcasing the parts and equipment that comprise the CsINPG setup.

In the development of CsINPG, a unique nozzle arrangement was added to the spinning vessel, as shown in Figure 1, where nozzles are inserted in four different areas around the pot. This pot features two separate nozzles, connected to the core chamber and the sheath chamber, both of which are constructed from 316 stainless steel tubing. However, the primary difference from previous manufacturing processes is that both nozzles protrude from the external shell of the vessel. Previous core–sheath manufacturing processes utilized a single nozzle connected to the core chamber, with an orifice surrounding the nozzle connected to the sheath chamber.^19^ These nozzles facilitate the controlled extrusion of the stock solution from the chambers and the formation of jet streams. These extruding jet streams are then collected using a water bath. The presence of a water bath significantly enhances the polymer choice and facilitates the use of “green polymers” such as cellulose and alginate for fiber manufacture. The water bath is essential as it enables the conversion of soluble solution jet streams to insoluble fibrous structures through the presence of coagulants. This is observed by the use of CaCl_2_ in the water bath when using Na-Alg as the primary polymer in the stock solution. The coagulation bath used in this laboratory setup was a shallow Pyrex glass dish containing 500 mL of solution made with deionized water and 3.5% CaCl_2_. The principle of utilizing CaCl_2_ in the coagulation bath was to facilitate ion exchange, as the introduction of Na-Alg into the bath containing CaCl_2_ results in the Na-Alg’s sodium ions (Na^+^) being substituted by calcium ions (Ca^2+^) present in the CaCl_2_ solution of the coagulation bath. This substitution is followed by a cross-linking process, where the two positive charges on the Ca^2+^ ions link with negatively charged sites on alginate, forming ionic bridges between two alginate chains in an “egg box structure”, resulting in the formation of insoluble calcium alginate fibers.

### 2.3 Production Variables

#### 2.3.1 Solution Parameters

To determine the optimum concentration, a wide variety of Na-Alg concentrations were tested, with characterizability assessed through fiber morphology analysis and overall solution spinnability. These solutions were formed, mechanically stirred, and then placed on a magnetic stirrer for 48–72 h to ensure efficient homogenization and mixing of Na-Alg in the solution. The viscosity was measured using the Brookfield DV-III Ultra rheometer, and the surface tensions were calculated using the Kruss tensiometer.

#### 2.3.2 Vessel Rotation Speed

Based on the analysis of previous gyration practices and trials with varying rotational speeds, a speed of 11,000 rpm was chosen as the optimum. A DC motor connected to the spinning vessel generated the spinning speed, which in turn produced the primary centrifugal force required for CsINPG. Speeds above the optimum produced short, broken fibers due to the kinetic instability of jet streams, while speeds below the optimum resulted in inefficient fiber formation.

### 2.4 Imaging

The fibers produced were analyzed and examined using various advanced characterizing techniques. Scanning electron microscopy (SEM) was performed using the ZEISS Gemini SEM 360. Prior to examination, the fibers were gold-coated to enhance conductivity using the Leica ACE 600 Sputter coater. The SEM analysis allowed the production of clear, detailed images of the fibers’ morphology and character. ImageJ (software) was used to accurately measure the diameter and size of the fibers using the images generated during SEM. Confirmation of the accurate functioning of the spinning vessel and proof of core–sheath fiber formation were obtained using confocal microscopy with the LSM 710 confocal microscope.

## 3 Results and Discussion

### 3.1 Polymer Choice

Na-Alg was chosen as the primary polymer over other green and nongreen polymers, such as chitosan, cellulose, polylactic acid (PLA), and other starch-based polymers, due to its range of superior advantages for the formation of core–sheathed fibers in this study, as shown in Table 1. Na-Alg used in this study is a derivative of natural alginate, which is found in the cell walls of brown seaweed and certain types of algae. The process begins with the harvesting of brown algal species such as *Laminaria* and *Ascophyllum*, which are rich in alginic acid.^23^ The harvested seaweed is washed, dried, ground, and treated with dilute alkaline solutions, such as Na_2_CO_3_,^24^ which results in dissolution. Alternatively, another method is precipitation using HCl and neutralization with NaOH. Following both methods, the insoluble residues are filtered out, and the final solution is purified through repeated filtration. It is then concentrated, spray-dried, or precipitated to form Na-Alg powder.

**Table 1:** Comparative Analysis of the Potential Polymers for Fiber Manufacture with Considerations on Their Sources, Renewability, Sustainability, Biocompatibility, and Industrial Maturity

polymer type	source	renewability	biodegradability	biocompatibility	gel forming ability	industrial maturity
alginate	marine algae	renewable	excellent	excellent	excellent	niche (^21^)
starch based	food crops	renewable	good	good	negative	moderate (^22^)
PLA	plant sugars	renewable	moderate	good	negative	excellent (^23^)
chitosan	crustacean waste	renewable	good	excellent	good	moderate (^24^)
cellulose	plant biomass	renewable	good	good	conditional	good (^25^)
PCL	fossil fuels	nonrenewable	good but slow	excellent	negative	good (^26^)
PE	fossil fuels	nonrenewable	nonbiodegradable	poor	negative	excellent (^27^)
PVC	fossil fuels	nonrenewable	nonbiodegradable	poor	negative	excellent (^28^)

Therefore, as shown in Table 1, alginate is an extremely attractive polymer for fiber usage compared to other polymers due to its wide availability, renewability, excellent biocompatibility, and biodegradability, which are superior to those of other widely used polymers.

First, polycarbonate was chosen as the primary material for constructing the gyration vessel. This material offers numerous advantages over other materials, such as aluminum and acrylic, which have been utilized in the fabrication of previous devices. The polycarbonate is distinguished by its transparency, a quality that sets it apart from aluminum. This property facilitates the observation of stock loading and movement, which is of considerable utility in examining and observing novel manufacturing processes. In addition, polycarbonate is a comparatively attractive material for upscaling in mass-scale fiber manufacture for commercial and industrial applications due to its combination of lightness, cost-effectiveness, and superior machinability. Furthermore, polycarbonate is superior to acrylic (PMMA) due to its excellent impact resistance and durability. This is in contradistinction to the brittle characteristics of acrylic. This makes polycarbonate more suitable for constructing gyration vessels, as high pressures and centrifugal forces are often used.^29^

### 3.2 Vessel Performance

Similarly, in the gyration vessel design, the primary reason for using nozzles and replacing orifices is highlighted in Figure 2. Orifices were used in the past in various manufacturing processes, including core–sheathed pressurized gyration. However, there was limited flow control, variable fiber uniformity, low jet stability, limited customizability, and moderate reproducibility, which were all overcome through the replacement of the orifices with nozzles.^17^ However, in this novel design, there are protruding nozzles from both chambers, with the core chamber nozzle measuring 6 mm in length, while the sheath chamber nozzle measures 5 mm in length. The attachment of nozzles for both chambers and the replacement of orifices facilitates improved flow efficiency through flow streamlining, which enhances the overall flow characteristics of spray patterns, velocity profiles, and pressure drops, creating continuous, uniform fibrous structures. Additionally, the core chamber nozzle is extended by 1 mm to facilitate the earlier creation of jet streams from the core, ensuring the core stream is already formed when the sheath stream begins to form. This allows the coating of the core stream with the sheath stream, facilitating the formation of conformally coated core–sheathed fibrous material. These nozzles have an internal diameter of 0.35 mm, which was determined to be optimal through analysis of previous manufacturing processes. Diameters lower than 0.35 mm were found to be too thin, often leading to nozzle blockage and preventing efficient flushing and jet formation of the stock solution in the chambers. Similarly, wider diameters, such as 0.5 mm, were too thick and led to a decrease in flow stability and disrupted jet formation. This resulted in the choice of 0.35 mm as the optimum internal nozzle diameter, achieving a balance between fine jet formation, reduced nozzle blockage, and efficient solution flushing.

**Figure 2:**
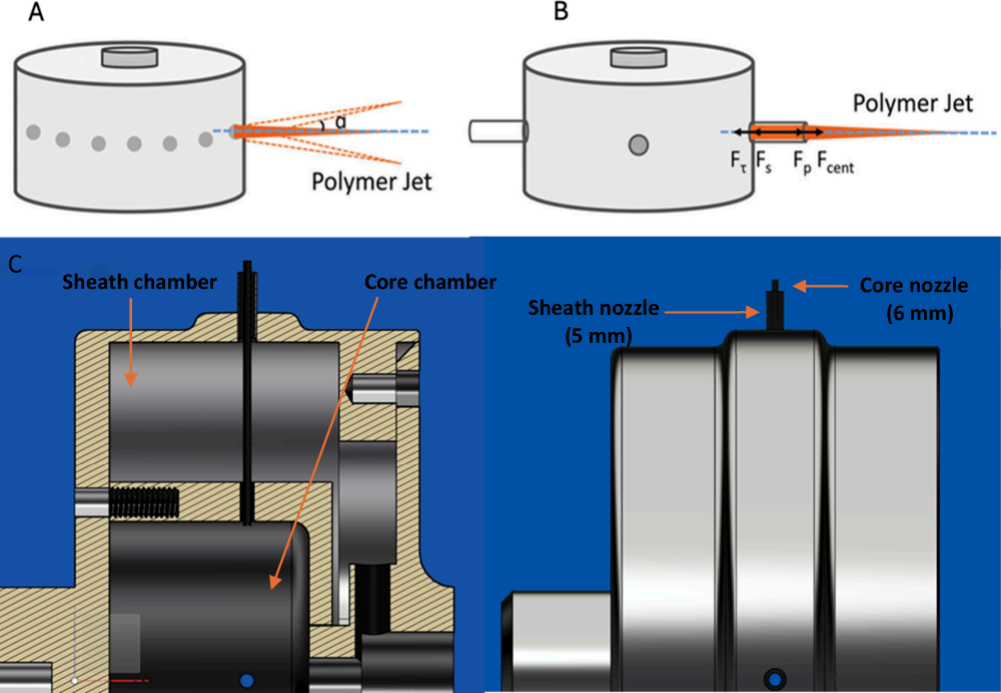
(^17^) Adapted with permission under a Creative Commons (CC BY 4.0) from ref 17 with permission from John Wiley and Sons. (A) Jet stream formation and solution ejection through an orifice. (B) Jet stream formation and solution ejection through a nozzle. (C) Labeled side profile highlighting the spinning vessel’s structures, compartments, and nozzle arrangement.

This process of stock solution ejection via the nozzles to form streaming jets can be represented through the Navier–Stokes Coriolis equation, which is a modification of the classical Navier–Stokes equation to consider the non-inertial frame of the spinning vessel and accounts for the Coriolis force, centrifugal force, pressure gradients, and the gravitational force exerted on the stock solution to convert the stock solution in the chambers to jet streams via the nozzles.^30^

$$\rho \left[\mathrm{\partial v}/\mathrm{\partial t}+\left(v\cdot \nabla \right)v+2\Omega \times v+\Omega \times \left(\Omega \times r\right)\right]=-\mathrm{\nabla P}+{\mu \nabla }^{2}v+\mathrm{\rho g}$$

where

- ρ = fluid density
- *v* = velocity vector of fluid (in the rotating frame)
- *t* = time
- Ω = angular velocity vector of the rotating system
- *r* = position vector from the axis of rotation
- *P* = pressure
- μ = dynamic viscosity
- *g* = gravitational acceleration vector
- 2Ω × *v* = Coriolis force
- Ω × (Ω × *r*) = centrifugal force
- ∇*P* = pressure gradient
- μ∇^2^ *v* = viscous (diffusion) term

On the left-hand side of the equation, (ρ­[∂*v*/∂*t* + (*v* × ∇)*v*]) accounts for how the velocity changes with time and how fluid velocity changes as it moves through space. Similarly, (+2Ω × (*v*)) accounts for the Coriolis force, which causes the curving of fluid movements and results in the formation of spiral jet trajectories. The (+Ω × (Ω × *r*)) component represents the centrifugal force, which pushes the stock solution radially outward toward the vessel walls and into the nozzles, facilitating the flushing of solution through the nozzles. On the right-hand side of the equation, −∇*P* represents the pressure gradient caused by the injected N_2_ gas, and this assists and facilitates the high-speed flushing and ejection of solution through the nozzles to form jets. The (+μ∇^2^ *v*) component accounts for the internal friction of the fluid and the resistance encountered during the fluid’s fast motion, especially near the nozzle walls. Finally, the (+ρ*g*) component highlights the gravitational pull of the jet due to the inverted axis of the setup, which pulls the jet downward into the coagulation and assists in shaping and settling the extruding jet streams.^31^ Therefore, this equation effectively explains the process by which the solution in the chambers is flushed from the nozzle to form jet streams, which are then converted into fibrous structures. This process occurs simultaneously in both chambers to form core–sheathed fibers.

### 3.3 Solution Parameters

The concentration of Na-Alg used in solution formation was the primary factor affecting overall solution viscosity and surface tension, which in turn influenced spinnability and fiber formation.

As shown in Table 2, an increase in Na-Alg concentration leads to an increase in viscosity. However, an optimum had to be found because solutions with viscosities below the optimum would be too runny, and solutions with viscosities greater than the optimum would be too dense and thick and would not be efficiently flushed from the chambers to form fibrous structures.

**Table 2:** Wide Variety of Na-Alg Concentrations Tested, along with the Corresponding Solution Viscosities and Surface Tensions, Considering the Standard Deviations (SD)

Na-Alg concentration %(w/w)	mean solution viscosity (mPa·s)	mean surface tension (mN/m)
0.5	339 ± 20	24.3 ± 1.0
1.0	563 ± 47	30.2 ± 1.0
1.5	2067 ± 199	37.9 ± 0.7
2.0	7900 ± 202	59.7 ± 2.8
2.5	14,696 ± 303	61.5 ± 2.9
3.0	31,902 ± 400	64.2 ± 1.6
3.2	48,648 ± 259	66.9 ± 2.5
3.5	71,634 ± 904	68.7 ± 1.9

The CsINPG process is easily customizable, user-friendly, and characterizable for unique fiber formation. This customizability is achieved through the wide variety of parameters that dictate fiber formation, such as solution viscosity, which is controlled by the Na-Alg concentration, pressure, speed, and collection distance between the bath and the nozzle tips. The optimal values for each parameter, along with the method used to obtain these results, are highlighted below.

Figure 3 illustrates the significant effect of an increase in Na-Alg concentration, along with the corresponding changes in average fiber diameter and their impact on fiber morphology. Solutions with Na-Alg concentrations from 0.5 to 1.5% formed films and fibrous films, while 2–3% formed flat, ribbon-like fibers and 3.5% formed massive fibrous trunks with large fiber diameters. Therefore, based on solution spinnability, fiber morphology, average fiber diameters, and previous published information,^15^ a compromised Na-Alg concentration of 3.2% was chosen as the optimum Na-Alg concentration. This optimum produced fibers with the most ideal morphology, featuring the finest diameters, shape, alignment, and uniformity, as shown in Figure 3G. Furthermore, a clear co-relation is visible between Na-Alg concentration and average fiber diameter, where an increase in Na-Alg concentration until the optimum resulted in a decrease in fiber diameter with the optimum Na-Alg concentration of 3.2% producing the fibers with the lowest fiber diameters and an increase in Na-Alg concentration beyond the optimum increased fiber diameter, which is portrayed through the graphical representation of Figure 3. This is evident in the graphical representation of Figure 3, which illustrates how an increase in Na-Alg concentration, up to the optimum, leads to a reduction in fiber diameter, as indicated by the downward-sloping curve. However, beyond the optimum (3.2%(w/w) Na-Alg), the average fiber diameter begins to increase again, as shown by the upward-facing curve.

**Figure 3:**
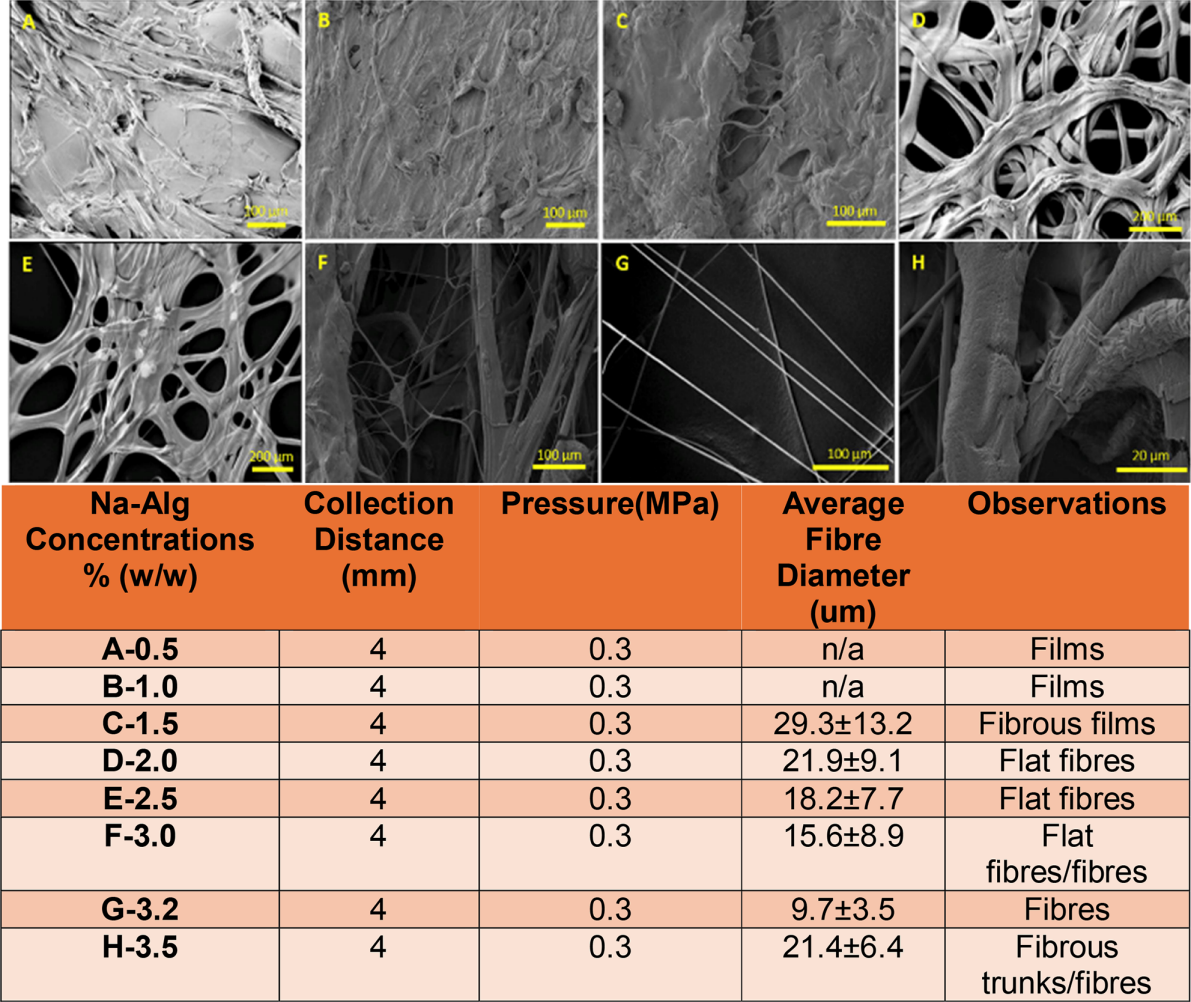
Detailed figure showcasing the effect of varying Na-Alg concentrations on fiber morphology and diameters. For the 0.5 and 1% Na-Alg concentrations, the fiber diameters are at zero because it was not possible to calculate the fiber diameters of the films.

Therefore, 3.2% was chosen as the optimum, as it produced the fibers with the finest average fiber diameters­(<10 μm) and the fibers with the most desirable morphology in comparison to the films or the flat, ribbon-like structures produced by Na-Alg concentrations below the optimum or the fibrous trunks produced by Na-Alg concentrations above the optimum. Additionally, this highlights that the optimum Na-Alg concentration yields the best viscoelasticity, resulting in the most optimal solution spinnability character.

### 3.4 Collection Distance

Similarly, another parameter affecting fiber morphology is the air gap or the collection distance between the nozzle tips and the surface of the solution in the water bath. The primary principle for determining the optimum collection distance was to find the ideal distance for achieving balanced elongation and stretching. The optimal values were calculated through multiple experiments, as shown in Figure 4.

**Figure 4:**
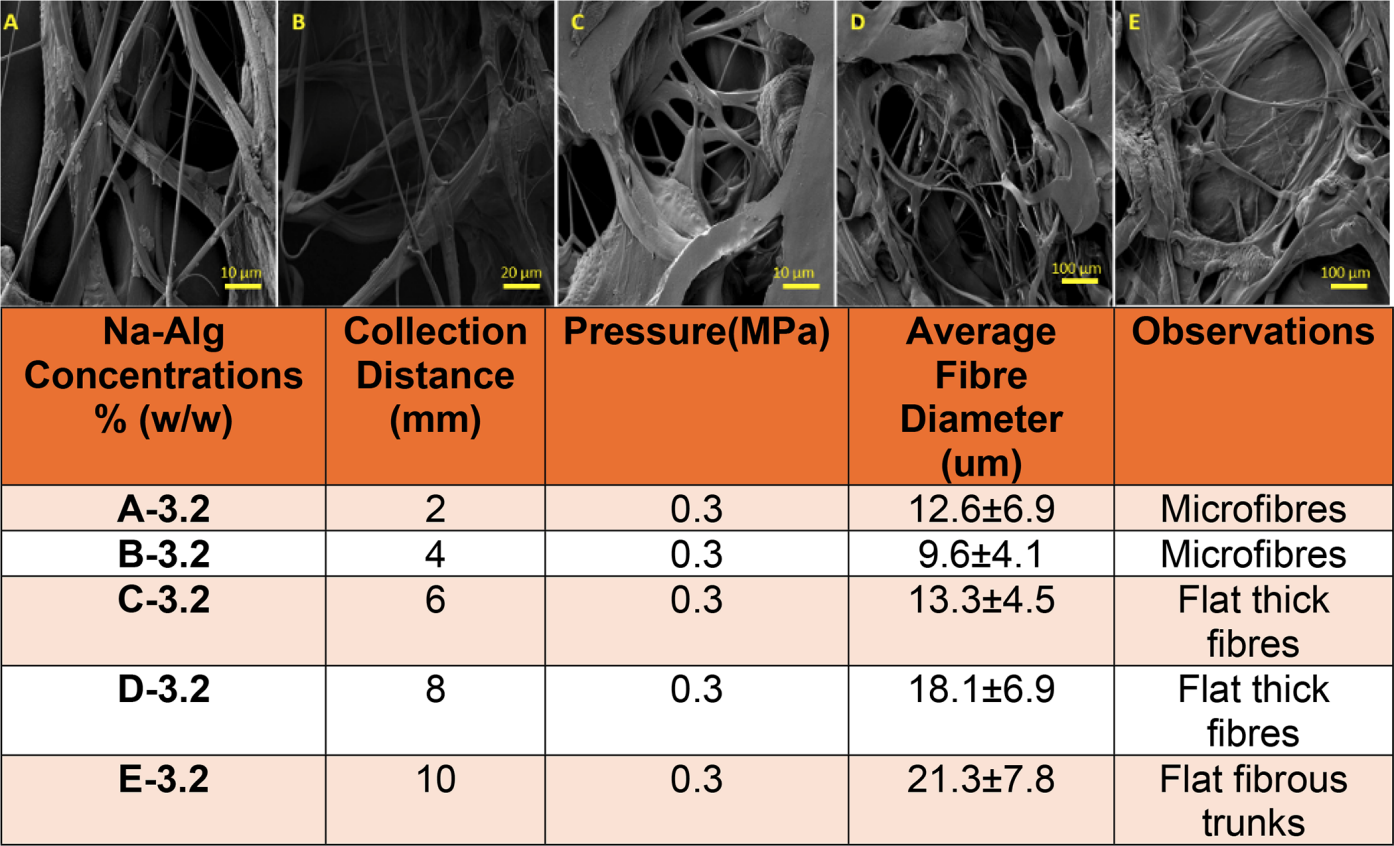
Detailed figure showcasing the effect of varying fiber collection distances on fiber morphology and average fiber diameters.

The detailed information in Figure 4 highlights the effects of collection distance on fiber diameter and morphology, where an increase in collection distance up to the optimum results in a reduction in average fiber diameter, and beyond the optimum, an increase in fiber diameter is observed. Based on the data collected, 4 mm was deemed the optimum as it produced the fibers with the best character and finest fiber diameters, as observed in Figure 4B. The fiber character and diameter for collection distances of 2 and 4 mm were very similar; however, 4 mm was chosen because air gaps of 4 mm or less between the nozzle tip and the water surface resulted in excessive turbulence and splashing, which caused the loss of a large amount of solution in the water bath and disrupted fiber formation. Furthermore, analysis of fiber morphology showed that 4 mm produced microfibers due to optimum jet stream stretching and elongation; however, beyond this, flat, thick fibrous structures and trunks formed due to excessive stretching, elongation, relaxation, and jet stream aggregation. Therefore, based on these factors, 4 mm was chosen as the optimum fiber collection distance between the nozzle tip and the solution surface in the water bath.

### 3.5 Pressure

The pressure applied to the spinning vessel during fiber formation via the N_2_ gas cylinder significantly affects and determines fiber character, as pressure differentials above the optimum can result in excessive kinetic energy of jet streams, leading to jet stream instability and a lack of stretching time, which in turn leads to fiber aggregation and the formation of fibrous trunks. Additionally, due to excessive kinetic energy, there may be jet stream breakage, leading to shortened or damaged fibers. Pressures below the optimum would result in inefficient fiber formation due to inefficient solution ejection, which would also reduce the jet streams’ stretching, increasing average fiber diameters. Varying pressures were tested to find the optimum, as shown in Figure 5.

**Figure 5:**
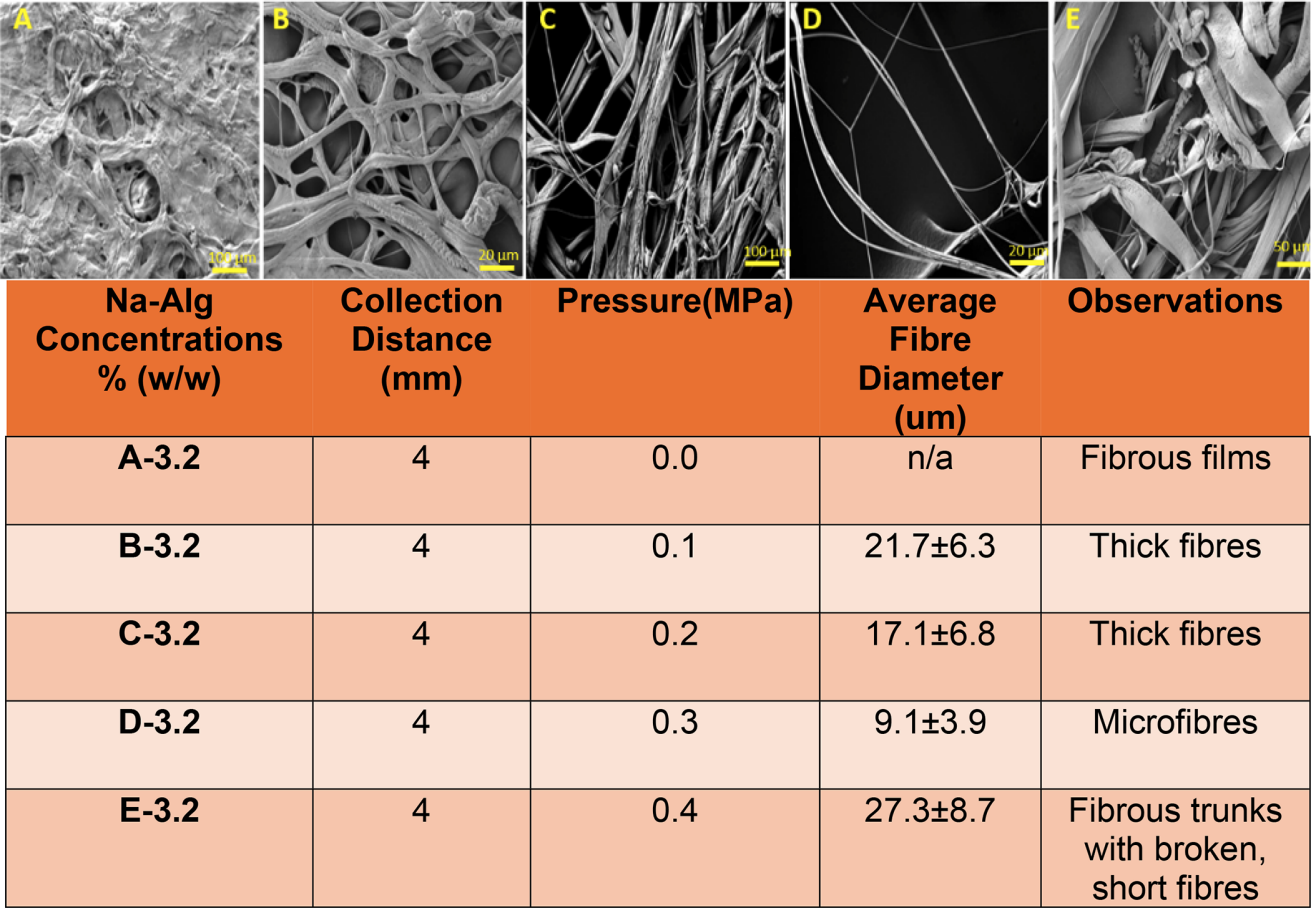
Effect of varying the pressure differentials on fiber morphology and average fiber diameters.

The detailed information in Figure 5 shows the effects of varying pressure on fiber morphology and average fiber diameters. The average fiber diameter for 0 MPa was not calculated as there was insufficient fiber ejection through the nozzles, and the small patches of structures produced were film-like and had very few proper fibers, making measurements difficult. However, an increase in pressure until the optimum, which was 0.3 MPa, resulted in a decrease in fiber diameter and an improvement in fiber morphology due to the movement toward a compromised balance between kinetic energy and the relaxation/stretching of fibers, which results in reduced fiber aggregation and formation of fibers with fine diameters. Pressures above the optimum result in the formation of fibrous trunks with very large diameters, as observed in Figure 5 and the presence of short, broken fibers due to excessive kinetic energy and instability of jet streams.

### 3.6 Products

Based on these optimum values obtained, various batches of core–sheathed fibers were produced. First, proof of core–sheathed fiber production and the functionality of the spinning vessel were obtained through confocal microscopy analysis. For this examination, 1% Rhodamine B (C_28_H_31_ClN_2_O_3_) was infused with 3.2% Na-Alg to add a pink fluorescence to the solution. Similarly, 1% acriflavine hydrochloride (C_14_H_14_ClN_3_) was infused with 3.2% Na-Alg to add a yellow-orange tint to the solution. The acriflavine hydrochloride + Na-Alg solution was used in the core. The Rhodamine B+ Na-Alg solution was used in the sheath, which was necessary during confocal imaging to distinguish between the core and the sheath through spectral multiplexing.

The labeled confocal microscopy image in Figure 6A clearly shows the encapsulation of the core by the surrounding sheath. This is proof of the production of core–sheathed fibers and the efficient functionality of the novel CsINPG pot and technology. The development of these core–sheathed fibers offers multiple advantages over single-layer fibers. First, the core–sheath fiber configuration enables the independent modulation of mechanical and chemical properties by selecting different core and sheath materials. This facilitates the possibility of fine-tuning features such as mechanical strength, biodegradability, elasticity, and chemical reactivity, thereby enhancing overall fiber durability and structural integrity. Similarly, this core–sheath arrangement facilitates the controlled release of encapsulated substances, which is crucial during tissue engineering, drug delivery, and encapsulation systems. The encapsulation of active agents within the core controls the permeability of the sheath, allowing for sustained or stimuli-responsive release profiles, which are difficult to achieve with homogeneous fibrous systems and structures. This compartmentalization ensures spatial and temporal control over agent delivery, which reduces toxic burst releases and improves the efficacy of treatments. Furthermore, the presence of a distinct sheath layer facilitates surface functionalization without altering the inner payload or compromising the mechanical properties of the core. Surface modifications for unique applications such as antibacterial activity, hydrophilicity, or electrical conductivity can be applied to the sheath, while the core remains unchanged, preserving its intrinsic function. This approach enhances the overall functional flexibility of the fibers, enabling application-specific tailoring of fibers.

**Figure 6:**
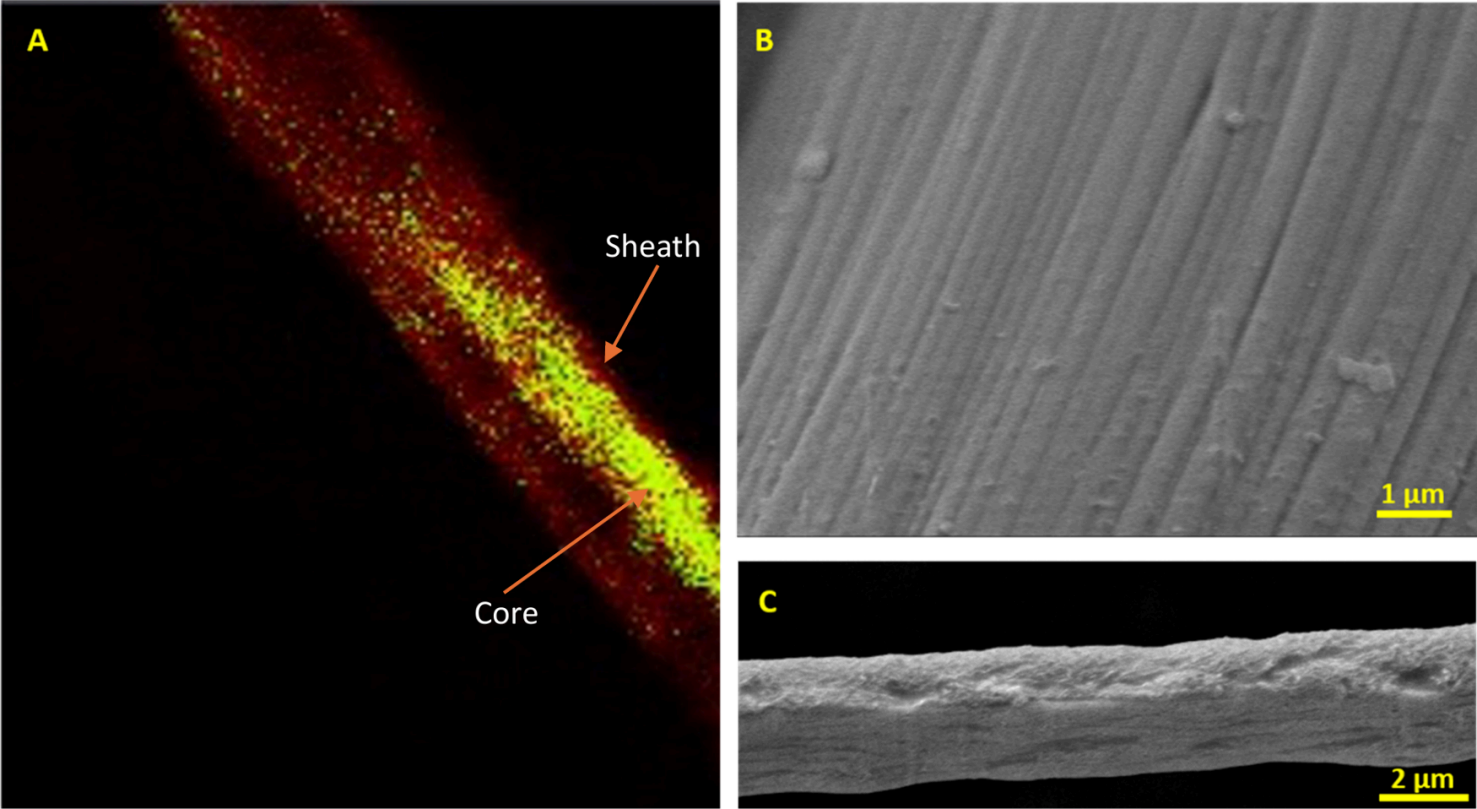
(A) Labeled confocal microscopy image. (B) Close-up SEM image of a fiber, highlighting the fiber uniformity. (C) Close-up SEM image, showcasing the unique texture of the fibers produced.

Additionally, the fiber surface depicted in Figure 6B shows the longitudinal uniformity, characterized by the consistently parallel striations running along its length. This highlights the controlled manufacturing process producing well-processed natural fibers and the capability of the fiber fabrication process in maintaining uniformity, which is useful for enhancing mechanical performance and for use in biological tissue scaffolds.

Furthermore, the fibers produced during CsINPG have a unique texture, as observed in Figure 6C, with a series of longitudinal grooves and ridges, resulting in a distinctive, rough, and striated appearance. These grooves are formed due to the uneven biphasic diffusion of coagulant, resulting from the viscosity differences between the Na-Alg solution and the Ca^2+^ ions in the coagulation bath. This unique morphology enhances the fiber’s advantages by significantly increasing its surface area through improved interfacial interactions, which are crucial in applications such as drug delivery, filtration, and cell adhesion.^32^

Moreover, the optimization of the parameters required for fiber fabrication is essential, as it controls the customization of the entire fabrication process but also affects fiber morphology and size. The synergistic balance of all these parameters is critical for efficient fiber formation. This synergy is observed when Na-Alg solutions spun without pressure failed to overcome their surface tension efficiently and remained confined within the chamber. This scenario indicates the significant effect of high working pressure applied during CsINPG and its effectiveness in processing highly viscous solutions. An increase in pressure differentials results in an increase in the kinetic energy of the spinning jet, facilitating optimal stretching and relaxing of jet streams. Additionally, an increase in kinetic energy through higher working pressure reduces the time required for jet formation, thereby improving overall fabrication efficiency. Likewise, the rotational speed, controlled through the attached DC motor, influences the motion state and formation of jet streams because rotational speeds and the centrifugal force have to overcome the critical value to be ejected through the nozzles to form spinning jet streams.^33^ The critical value refers to the minimum rotational speed at which significant physical effects, such as particle separation or phase transition, begin to occur within a system.^34^ However, low speeds (<9500 rpm) could result in insufficient kinetic energy of jet streams, leading to decreased fiber production and reduced system efficiency. Similarly, an optimum air gap is essential to achieving stretching through dry jet formation and relaxation; however, it should be balanced to prevent excessive elongation and relaxation. Finally, an optimum Na-Alg concentration is vital too. This is the primary component controlling the interplay between fiber formation and fluid dynamics, as an optimally viscous solution ensures sufficient polymer chain entanglement, which is essential for the generation of continuous fiber formation. If the Na-Alg concentration and the viscosity are too low, then the solution would lack the cohesive strength required to sustain jet elongation, leading to the formation of beads or discontinuous fibers. Likewise, excessively high Na-Alg concentrations and viscosities can hinder nozzle flow, resulting in irregular jetting or nozzle blockage, which in turn affects productivity and uniformity. Therefore, the synergistic combination of the parameter optimums Na-Alg concentration of 3.2%, 0.3 MPa, 11,000 rpm, and an air gap of 4 mm, ensures optimum fiber manufacture and morphology.

Likewise, the selection of a suitable polymer for fiber manufacture plays a crucial role in determining the mechanical, physicochemical, and environmental characteristics of the final fibrous structure, as observed in Table 1. Alginate is a highly advantageous primary polymer for use in CsINPG due to its unique structural and processing characteristics. Alginate is a naturally occurring anionic polysaccharide composed of β-d-mannuronic acid (M) and α-l-guluronic acid (G) residues, arranged in homopolymeric (M–M or G–G) and heteropolymeric (M–G) blocks. This block structure significantly influences its mechanical performance, gelation behavior, and ion exchange properties. Alginate’s most prominent advantage is its ability to form fibers through ionic cross-linking, which occurred in this study with divalent cations to create an “egg box” structured model.^35^

In this study, Na-Alg was streamed into the water bath containing a solution of 3.5% calcium chloride. This leads to a chemical reaction and the formation of calcium alginate fibers, where the Na-Alg’s two central sugar units (mannuronic acid/M-blocks and guluronic acid/G-blocks) interact with the calcium chloride solution and leads to the Na^+^ ions of alginate being replaced by Ca^2+^ from the water bath. The +2 charge of the divalent cations facilitates the simultaneous binding to two separate alginate chains and results in the formation of the “egg box” model. In this model, the guluronic acid of adjacent alginate chains align to form cavities or pockets, where calcium ions fit. This arrangement resembles the fitting of eggs into the walls of an egg box, resulting in the naming of this arrangement as the egg box model. This 3D cross-linking network and grid-like binding of calcium ions provides the strength, structure, and firm formation required for efficient fiber formation.^37^ Therefore, the rapid gelation characteristic of Na-Alg facilitates the use of mild, environmentally friendly solvents, such as water, and eliminates the need for organic solvents or thermal processing typically required with synthetic polymers. This improves process safety and reduces the overall carbon and toxicological footprint of fiber production. Additionally, Na-Alg is highly biocompatible and biodegradable, as its enzymatic degradation under physiological conditions produces nontoxic monosaccharides. This character contrasts with synthetic polymers, which are typically nonbiodegradable and accumulate in the environment.^36^ Furthermore, the degradation kinetics of alginate fibers can also be controlled through the alteration of the M/G ratio and degree of cross-linking, thereby facilitating tailored stability during use and postdisposal degradation. Moreover, alginate’s abundant hydroxyl and carboxyl groups facilitate hydrophilicity and high moisture retention.^37^ This enables excellent water uptake and swelling properties, improving fiber softness and enhancing user comfort. Moreover, alginate is abundantly renewable, as it is derived from brown algal species and the extraction process requires low energy and does not rely on petroleum-based feedstocks, making it environmentally sustainable and having a very low opportunity cost associated with its usage.^38^ Finally, alginate’s current niche commercial usage enables businesses to explore its various applications, facilitating enterprises to enter and exploit markets with previously unexplored customers and reduced competition. Therefore, this highlights in depth the multiple benefits of alginate and the reasons for its choice as the primary polymer in fiber manufacture.

These manufactured core–sheathed alginate fibers have various uses. First, these fibers can be extensively utilized in the biomedical field, especially for various biomedical applications. Additionally, core–sheathed fibers can be used for the control and treatment of advanced wound care. The alginate surrounding sheath of fibers plays a crucial role in creating a moist wound environment, which is essential to effective healing. Upon contact with wound secretions, the calcium alginate in the sheath ionically exchanges with sodium ions from the wound exudate, forming a hydrogel that conforms to the wound bed, facilitating autolytic debridement, and supports cellular migration and tissue regeneration.^39^ This biomedical efficacy can be further improved by engineering the fiber core to carry antimicrobial agents, such as silver nanoparticles, antibiotics, anti-inflammatory compounds, or natural plant phytochemicals, thereby facilitating sustained and localized release and enhancing overall therapeutic efficacy. This would make these fibers optimum for use and managing chronic wounds such as diabetic foot ulcers, venous leg ulcers, and pressure sores, where infection control and prolonged drug delivery are crucial to prevent further infection.^40^ Similarly, core–sheathed alginate fibers produced by CsINPG offer a highly controllable platform for targeted drug delivery and release. The core may encapsulate hydrophobic or hydrophilic drug molecules, while the alginate sheath acts as a diffusion barrier modulating drug release kinetics. By adjusting the cross-linking density of the alginate or incorporating responsive elements into the sheath, release can be triggered by specific stimuli such as pH, temperature, or enzymatic activity. This makes core–sheathed alginate fibers suitable for oral drug delivery systems where the drug needs to be protected from stomach acid and released in the intestine to facilitate complex composite release systems,^41^ or for implantable drug delivery devices where sustained release over long periods is required, such as during the treatment of cancer^42^ or chronic inflammatory conditions.^43^ Furthermore, core–sheathed alginate fibers can be significantly useful in tissue engineering and regenerative medicine. These fibers have the potential to function as biomimetic scaffolds that support cell adhesion, proliferation, and differentiation. The alginate sheath provides a soft, hydrated, and ionically conductive microenvironment, similar in composition and structure to the natural extracellular matrix (ECM). At the same time, the core may include bioactive molecules, collagen, or stem cells to promote tissue regeneration.^44^ An example of this is the use of core–sheathed fiber in neural tissue engineering, where the fiber core can be loaded with neurotrophic factors, while the alginate sheath provides protection and directional guidance for axonal growth.^45^ Such scaffolds can also be used for cartilage repair, skin regeneration, and vascular tissue engineering, where both mechanical integrity and biological functionality are required.^46^ Additionally, for applications in environmental science, core–sheathed alginate fibers are being explored for use in water purification and pollutant adsorption. The alginate sheath can be functionalized with chelating groups that selectively bind to heavy metals such as lead, cadmium, and mercury. Meanwhile, the fiber core may consist of activated carbon, zeolites, or other adsorptive materials that enhance the overall binding capacity. This core–sheath arrangement and its unique morphology facilitate a high surface contact area with contaminants while protecting the active core from mechanical degradation. Furthermore, due to the biodegradable nature of alginate, these fibers offer a green and sustainable solution for large-scale water treatment applications.^47^ Moreover, core–sheathed alginate fibers produced using CsINPG can be used in the field of smart textiles and bioelectronics, with a growing interest being observed in the fibers’ potential to integrate sensing and actuation functionalities. Through the incorporation of conductive materials, such as carbon nanotubes, graphene, or metallic nanoparticles, into the core, these fibers can function as flexible, stretchable sensors capable of detecting mechanical deformation, temperature, or even biochemical markers in sweat or interstitial fluid.^48^ Furthermore, the alginate sheath not only provides a protective and biocompatible interface with the skin but also can respond to environmental stimuli, enabling dynamic control over sensor sensitivity. These properties are especially valuable in the development of wearable health-monitoring devices and transient electronics, where materials must be both functional and safe for long-term human use.^49^

## 4 Conclusions

The successful development of core-sheathed alginate fibers via CsINPG presents a noteworthy advancement in the field of functional and responsive fiber fabrication. This novel manufacturing technique enables precise construction of core-sheath fiber architectures, facilitating the encapsulation of diverse core materials within a structurally stable and biocompatible alginate sheath. The resulting fibers exhibit a high degree of versatility, offering multifunctionality suited to a wide range of scientific and industrial applications. The study has highlighted the broad utility of CsINPG-fabricated fibers in several key domains, with an application focus on biomedical engineering, environmental applications, and sustainability, as well as in high-tech, advanced fields such as smart textiles and biosensor applications. Therefore, CsINPG represents a robust and innovative platform for core-sheath fiber fabrication, with its adaptability. The multifunctional potential of the resulting fibers, combined with this adaptability, positions it as a valuable tool for future developments in tissue engineering, environmental sustainability, and wearable technologies. Continued research into the optimization of CsINPG parameters, material combinations, and application-specific performance will further enhance its impact across scientific and technological domains.

## Supplementary Material



## Notes

CsINPG process (MP4)
